# Varespladib (LY315920) and Methyl Varespladib (LY333013) Abrogate or Delay Lethality Induced by Presynaptically Acting Neurotoxic Snake Venoms

**DOI:** 10.3390/toxins12020131

**Published:** 2020-02-20

**Authors:** José María Gutiérrez, Matthew R. Lewin, David. J. Williams, Bruno Lomonte

**Affiliations:** 1 Instituto Clodomiro Picado, Facultad de Microbiología, Universidad de Costa Rica, San José 11501-2060, Costa Rica; bruno.lomonte@ucr.ac.cr; 2 Ophirex, Inc., Corte Madera, CA 94925, USA; matt@ophirex.com; 3 California Academy of Sciences, San Francisco, CA 94118, USA; 4 Global Snakebite Initiative, Ashgrove Qld 4060, Australia; dr_davidwilliams@outlook.com

**Keywords:** Varespladib, LY315920, LY333013, phospholipase A_2_, neurotoxicity, lethality

## Abstract

The phospholipase A_2_ (PLA_2_) inhibitor Varespladib (LY315920) and its orally bioavailable prodrug, methyl-Varespladib (LY333013) inhibit PLA_2_ activity of a wide variety of snake venoms. In this study, the ability of these two forms of Varespladib to halt or delay lethality of potent neurotoxic snake venoms was tested in a mouse model. The venoms of *Notechis scutatus*, *Crotalus durissus terrificus*, *Bungarus multicinctus,* and *Oxyuranus scutellatus*, all of which have potent presynaptically acting neurotoxic PLA_2_s of variable quaternary structure, were used to evaluate simple dosing regimens. A supralethal dose of each venom was injected subcutaneously in mice, followed by the bolus intravenous (LY315920) or oral (LY333013) administration of the inhibitors, immediately and at various time intervals after envenoming. Control mice receiving venom alone died within 3 h of envenoming. Mice injected with *O. scutellatus* venom and treated with LY315920 or LY333013 survived the 24 h observation period, whereas those receiving *C. d. terrificus* and *B. multicinctus* venoms survived at 3 h or 6 h with a single dose of either form of Varespladib, but not at 24 h. In contrast, mice receiving *N. scutatus* venom and then the inhibitors died within 3 h, similarly to the control animals injected with venom alone. LY315920 was able to reverse the severe paralytic manifestations in mice injected with venoms of *O. scutellatus, B. multicinctus,* and *C. d. terrificus.* Overall, results suggest that the two forms of Varespladib are effective in abrogating, or delaying, neurotoxic manifestations induced by some venoms whose neurotoxicity is mainly dependent on presynaptically acting PLA_2_s. LY315920 is able to reverse paralytic manifestations in severely envenomed mice, but further work is needed to understand the significance of species-specific differences in animal models as they compare to clinical syndromes in human and for potential use in veterinary medicine.

## 1. Introduction

Snakebite envenoming is a neglected tropical disease that kills or maims hundreds of thousands of people every year, especially in impoverished rural communities of sub-Saharan Africa, Asia, Latin America, and parts of Oceania [1]. Owing to the heavy impact that this disease has on global public health, the World Health Organization (WHO) launched an integrated strategy to significantly reduce the burden of these envenomings earlier last year [2]. One of the four pillars of this global strategy is to “ensure that safe, effective, affordable, accessible treatments for snakebite envenoming are available to all the people who need them” [2].

The centerpiece in the therapy of snakebite envenoming is the timely administration of safe and effective antivenoms [1,3]. If well-designed and manufactured following good manufacturing practices (GMPs), antivenoms are quite effective and safe, especially for controlling the life-threatening systemic effects of envenomings [3]. Nevertheless, one drawback of antivenom therapy is that it needs to be provided by trained healthcare staff in appropriately equipped clinical facilities. This becomes an issue when people have to travel long distances to reach health posts, or when antivenoms are not available in rural facilities. An unmet need is for the development of novel therapies that could be administered in the field rapidly after the onset of envenoming [4], as well as to augment antivenom performance where specific toxins have become inaccessible to antivenom upon distribution into the tissues, but might be amenable to inhibition or dislodgement by smaller molecules. Promising developments in this area have emerged, including natural and synthetic inhibitors of snake venom metalloproteinases (SVMPs) [5,6], phospholipases A_2_ (PLA_2_s) [7], cytotoxins of the three finger toxin family [8,9], and α-neurotoxins [10], among others. 

A promising sPLA_2_ inhibitor candidate with characteristics potentially filling some of these unmet needs is Varespladib (LY315920) and its orally available form methyl-Varespladib (LY333013). These inhibitors were developed by the pharmaceutical industry for the treatment of inflammatory syndromes such as sepsis, rheumatoid arthritis, and for acute manifestations of cardiovascular disease [11,12]. These avenues were abandoned following phase II or phase III clinical trials for these diseases, but these inhibitors could be repositioned for the therapy of snakebite envenoming owing to their broad ability to inhibit snake venom PLA_2_s [7]. These enzymes are abundant and toxicologically relevant components in many snake venoms of high medical impact [13,14,15,16,17]. Following the initial description of Varespladib’s capacity to inhibit PLA_2_ activity of many snake venoms in vitro [7], further studies demonstrated its ability to abrogate lethality in mouse and pig models of envenoming by the venoms of the elapid snakes *Oxyuranus scutellatus* and *Micrurus fulvius*, respectively, whose neurotoxic effect is based predominantly on the action of presynaptically acting neurotoxic PLA_2_s [18,19]. Furthermore, Varespladib was effective in the inhibition of myotoxicity induced by crude venoms and isolated PLA_2_s of viperid and elapid species [20,21]. This drug was reported to inhibit the in vitro coagulotoxic effects of venoms of African spitting cobras [22].

Four crude venoms containing potent presynaptically acting neurotoxic PLA_2_s of differing quaternary structure were selected to test the ability of Varespladib to abrogate, delay, or reverse neurotoxic venom effects. The four venoms selected were: *Notechis scutatus*, *Bungarus multicinctus*, *Crotalus durissus terrificus*, and *Oxyuranus scutellatus*. Varespladib’s two forms, administered intravenously (i.v.) as a single rapid bolus of LY315920 (drug) or orally as LY333013 (prodrug) following experimental envenoming, abrogated or delayed lethality and reversed paralysis in three out of four venoms. 

## 2. Results

### 2.1. Estimation of LD_50_s

The values of LD_50_ of the venoms, in 18–20 g mice by the s.c. route of injection, were: *N. scutatus*: 4.5 µg (95% confidence limits: 1.8–7.1 µg); *C.d. terrificus*: 7.9 µg (95% confidence limits: 4.8–11.6 µg), and *B. multicinctus*: 3.1 µg (95% confidence limits: 1.3–5.2 µg). The value of LD_50_ of the batch of *O. scutellatus* venom used in this study had been previously estimated as 0.012 µg/g (95% confidence limits: 0.004–0.023 µg/g) [23], i.e., 0.24 µg per 20 g mice (95% confidence limits: 0.08–0.46 µg). 

### 2.2. Rescue Experiments

Mice received s.c. injections of doses of venoms corresponding to 3 LD_50_s for *N. scutatus*, *C.d. terrificus*, and *B. multicinctus,* and either 3 LD_50_s or 12 LD_50_s for *O. scutellatus*. Then, either LY315920 or LY333013 were administered using various schedules, as described in Materials and Methods. In the case of *N. scutatus*, co-administration or delayed administration of the inhibitors did not prolong the survival time of envenomed mice and both venom control group and groups treated with the drugs died within 2–3 h (Table 1). In contrast, when *N. scutatus* venom was pre-incubated with LY315920 prior to injection in mice, the lethal effect was significantly delayed; mice injected with venom alone died within 2–3 h, whereas those receiving venom pre-incubated with LY315920 died between 10 and 15 h.

When mice were injected with *C. d. terrificus* venom, control mice died within 3 h, whereas regardless of whether they received (a) LY315920 intravenously or (b) LY333013 orally at any time point, with or without redosing at 240 min, treated mice survived for 3 h and 6 h but were all dead at 24 h (Table 2).

Mice envenomed with *B. multicinctus* venom had a different response depending on whether they received LY315920 or LY333013. Venom control group animals all died within 3 h. When LY315920 was provided i.v., mice receiving (a) the drug immediately, or (b) an immediate dose of drug and a repeat dose at 240 min, survived at 3 h, but three out of four were dead by 6 h, and none of them survived by 24 h (Table 3). In contrast, when LY333013 was administered orally either (a) immediately, or (b) as an immediate dose with a repeat dose at 240 min, all of them survived at 3 h and 6 h, but not at 24 h (Table 4). For both LY315920 and LY333013 delayed administration for 60 min, with or without redosing at 240 min, resulted in survival at 3 h and 6 h followed by death at 24 h in all test animals (Table 3 and Table 4).

Control mice injected with three LD_50_s of *O. scutellatus* venom died within 6 h, whereas those receiving 12 LD_50_s died within 3 h. All mice injected with three LD_50_s of venom, followed by either i.v. LY315920 or oral LY333013 administered (a) immediately, or at (b) 30 min, (c) 60 min, (d) 90 min, or (e) immediately and again at 240 min after venom injection survived the 24 h observation period. When mice were challenged with 12 LD_50_s of this venom, those receiving the inhibitors immediately or at 30 min, 60 min, or immediately and again at 240 min survived for 24 h, with the exception of one mouse treated with LY315920 at 60 min (Table 5). When the administration of inhibitors was delayed for 90 min, mice survived at 3 h and 6 h, but were dead by 24 h (Table 5). Since the inhibitors provided full protection during 24 h in the case of *O. scutellatus* venom, the ID_50_ of LY315920 (i.v.), was estimated in conditions when the inhibitor was injected immediately after the s.c. injection of 12 LD_50_s of venom. In these conditions, the ID_50_ was 5.94 mg/kg (95% confidence limits: 4.36–9.46 mg/kg). All mice survived the 24 h observation period with a dose of inhibitor of 10 mg/kg.

### 2.3. Reversion of Paralytic Effects

**Figure 1:**
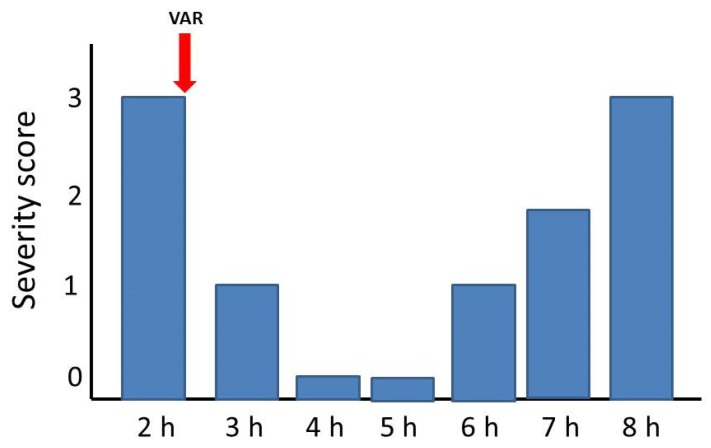
Reversion of paralytic manifestations in groups of four mice receiving three LD_50_s of *Crotalus d. terrificus* venom by the subcutaneous route. A single bolus dose of LY315920 (10 mg/kg i.v.) (VAR) was administered at the time indicated by the red arrow, when mice showed paralytic manifestations corresponding to a severity score of 3. Paralysis was reversed for the following 3 h and returned afterwards.

**Figure 2:**
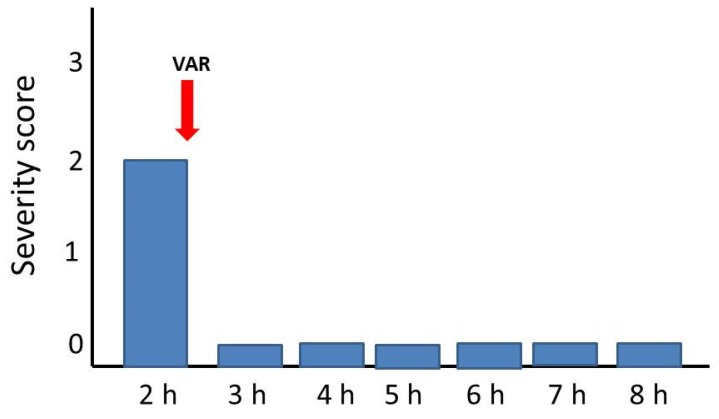
Reversion of paralytic manifestations in groups of four mice receiving three LD_50_s of *Bungarus multicinctus* venom. A single bolus dose of LY315920 (10 mg/kg i.v.) (VAR) was administered at the time indicated by the red arrow, when mice showed paralytic manifestations corresponding to a severity score of 2. Paralysis was completely reversed for the following 6 h.

**Figure 3:**
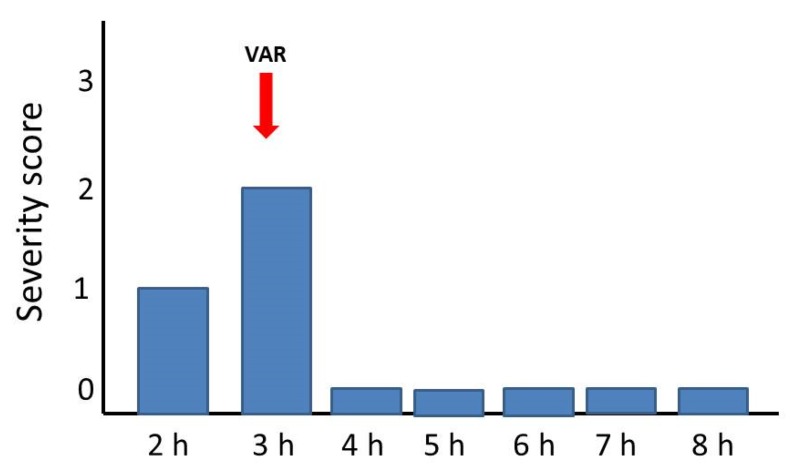
Reversion of paralytic manifestations in groups of four mice receiving 12 LD_50_s of *Oxyuranus scutellatus* venom by the subcutaneous route. A single bolus dose of LY315920 (10 mg/kg i.v.) (VAR) was administered at the time indicated by the red arrow, when mice showed paralytic manifestations corresponding to a severity score of 2. Paralysis was completely reversed for the following 5 h.

The ability of a single bolus dose of LY315920 (10 mg/kg) to reverse the manifestations of envenoming at a time when mice showed toxicity grades of 2 or 3 (see Materials and Methods for explanation) was assessed for the venoms of *C. d. terrificus*, *B. multicinctus,* and *O. scutellatus*. The venom of *N. scutatus* was not tested since the inhibitors were unable to delay lethality in rescue experiments. As shown in Figure 1, Figure 2 and Figure 3, there was a complete reversion of the paralytic manifestations caused by these three venoms. In the case of *C. d. terrificus* venom, paralysis recurred by 6 h and was severe by 8 h. In contrast, in the cases of *B. multicinctus* and *O. scutellatus* venoms, animals were devoid of paralytic manifestations for 8 h after treatment (Figure 2 and Figure 3).

### 2.4. Effect of Neostigmine

Administration of the anti-cholinesterase drug neostigmine and the anti-muscarinic drug atropine 5 min before *N. scutatus* venom injection did not have any effect on the toxicity of the venom, since envenomed mice receiving these drugs and those injected with PBS died at the same time span (2 to 3 h). Similar findings were observed in mice receiving neostigmine and atropine before envenoming, and LY315920 immediately after venom injection, as well as in mice receiving only LY315920 after envenoming. 

## 3. Discussion

The search for inhibitors that block the action of snake venom toxins, and which could be applied in the field rapidly after the onset of envenoming in a snake bite, has become an area of increasingly active research. In this regard, the search for inhibitors that have been developed for diseases whose pathogenesis is associated with endogenous enzymes, such as PLA_2_s and metalloproteinases, represents a potentially cost- and development time-efficient piggy-back approach by taking advantage of previous preclinical and clinical assessments of their safety (see for example [6,24]). Such is the case of the PLA_2_ inhibitor Varespladib (LY315920) and its orally available derivative methyl-Varespladib (LY333013), which were developed for the therapy of several unrelated conditions [11,12]. Recent studies have shown the ability of these drugs to inhibit enzymatic PLA_2_ activity of a large number of venoms in vitro [7], and to abrogate, in animal models, lethality induced by two neurotoxic elapid venoms whose toxicity is based on the presynaptic action of neurotoxic PLA_2_s [18,19].

In order to expand these observations, we have tested the efficacy of the two forms of Varespladib against venoms having presynaptically acting neurotoxic PLA_2_s of variable quaternary structure, as it is not known how differences in quaternary structure of PLA_2_s might affect their inhibition by Varespladib. *N. scutatus* venom contains notexin, a monomeric PLA_2_ [25,26,27]. β-bungarotoxin, present in the venoms of *Bungarus* sp, is a dimeric neurotoxin comprising a PLA_2_ subunit linked by disulfide bonds to a Kunitz-type proteinase inhibitor [28,29,30]. The venom of *C. d. terrificus* contains a high proportion of the dimeric neurotoxin crotoxin, comprising a PLA_2_ subunit linked, through non-covalent bonds, to an enzymatically inactive subunit [31,32]. *O. scutellatus* venom, in turn, contains the potent presynaptic toxin taipoxin, whose quaternary structure is characterized by three subunits, one of which is a catalytically active PLA_2_ [27,33]. 

We followed a rescue-type experimental protocol in which venoms were injected s.c., in the hind limb, and the inhibitors were administered at various time intervals either by the i.v. route (LY315920) or orally (LY333013), hence simulating a situation where the inhibitors are provided quickly and conveniently after a bite as they might be in a scenario where a person is bitten in a resource limited environment. In the cases of venoms of *N. scutatus*, *C. d. terrificus*, *B. multicinctus*, the selected dose of three LD_50_s induced lethality within 3 h, whereas in the case of *O. scutellatus*, mice receiving three LD_50_s died within 6 h. Therefore, with this venom we also used a higher challenge dose (12 LD_50_s) in order to shorten the survival time to 3 h, and also because this dose had been used in a previous study [18]. 

Administration of the inhibitors at the various schedules used resulted in a significant delay in the time of death in the cases of the venoms of *C. d. terrificus* and *B. multicinctus*, and in a complete protection during 24 h for the venom of *O scutellatus*, provided the inhibitors were administered at various time intervals after envenoming. These observations underscore the role of neurotoxic PLA_2_s in the overall toxicity of these three venoms. In the case of *B. multicinctus* venom, a puzzling observation was made when using LY315920, i.e., animals were protected when the drug was given 60 min after envenoming, but not when administered immediately upon venom injection. We hypothesize that this is due to a mismatch between venom toxicokinetics and drug pharmacokinetics. Since LY315920 has a short half-life, it is likely that, when administered at 0 min, its concentration in the body drops by the time β-bungarotoxin acts on neuromuscular junctions. Such phenomenon was not observed when LY333013 was administered orally, probably because the pharmacokinetics of this form of Varespladib differs, reaching inhibitory levels in the circulation at later time intervals.

In contrast with these three venoms, no protection was observed in the case of *N. scutatus* venom. One possibility to explain this finding has to do with the potential role of post-synaptically acting α-neurotoxins present in this venom [34,35]. Our observations with the inhibitor neostigmine, however, do not support this hypothesis, since administration of this anticholinesterase drug did not inhibit or delay lethality by *N. scutatus* in our experimental setting. An alternative explanation emerges from the observation that LY315920 is able to significantly delay the lethality when incubated with the venom before injection, hence supporting the view that neurotoxic PLA_2_s, such as notexin, are indeed responsible for this venom’s toxicity. Hence, the lack of efficacy of Varespladib when injected after the onset of envenoming could be related to a mismatch of neurotoxic PLA_2_ toxicokinetics and LY315920 pharmacokinetics. These are issues of practical and academic interest that deserve further investigation. The complexity of snake venom-induced neurotoxicity [36], the species-to-species variation in the presence of PLA_2_ neurotoxins, and the toxicokinetic/pharmacokinetic relationships demand innovation and attention in detail in the methods of preclinical assessment of efficacy of toxin specific inhibitors for different snake venoms.

The observation, in the venoms of *C. d. terrificus* and *B. multicinctus*, that lethality was not completely prevented but only delayed might have also to do with the mismatch between the pharmacokinetics of the inhibitor and the toxicokinetics of the neurotoxins, since the former is likely to have a shorter half-life than the latter. These drawbacks can be solved by implementing a treatment protocol based on continuous rate infusion of the inhibitor at lower doses, with or without initial bolus administration, as demonstrated in a pig model of envenoming by the venom of the coral snake *Micrurus fulvius* [19]. Similarly, studies of these types of molecules, with and without antivenom, administered at different times and orders of administration should shed light on the most effective therapeutic protocols. 

The recovery from severe neurotoxic paralysis of envenomed mice by the i.v. administration of LY315920 is noteworthy. Studies on the mechanism of action of presynaptically acting snake venom neurotoxic PLA_2_s have shown drastic ultrastructural alterations at the nerve terminal, hence explaining the observed lack of efficacy of therapeutic antivenoms when administered after the onset of neurotoxicity [29]. Our findings underscore that Varespladib is able to reverse neurotoxic manifestations in the cases of *C. d. terrificus*, *B. multicinctus*, and *O. scutellatus* venoms. In the case of *O. scutellatus* venom, it abrogates toxicity even at a time when antivenom is largely ineffective in a mouse experimental model [18]. The basis of these intriguing observations is unclear at present, but suggests that this inhibitor has significant therapeutic potential in neurotoxic snakebite envenomings, even after the onset of paralysis, and could become a useful experimental tool to further understand the molecular and cellular basis of PLA_2_-induced neurotoxicity. 

It has been proposed that neurotoxic venom PLA_2_s initially bind to a receptor at the nerve terminal, a step followed by enzymatic hydrolysis of phospholipids in the plasma membrane of the terminal. This in turn causes a calcium influx that promotes the release of the neurotransmitter stored in synaptic vesicles and the increment in the membrane permeability to ions which, in the particular case of calcium, causes the degeneration of the nerve terminal and the irreversible blockade of the transmission [37]. Other studies have provided evidence for an internalization of these neurotoxins into the nerve terminal cytoplasm, with the possible binding to intracellular receptors and the consequent toxicity secondary to hydrolysis of membranes of organelles, such as those of mitochondria [38]. It is possible that these mechanisms are not mutually exclusive, depending on the toxin concentration at the synaptic site, i.e., a threshold phenomenon. 

In this mechanistic scenario, the observed ability of LY315920 to reverse paralytic manifestations may be based on (a) the ability of the inhibitor to dissociate the binding of the toxins to their membrane receptor, owing to the high affinity of Varespladib for the PLA_2_s, before extensive phospholipid hydrolysis has occurred and prior to the internalization of the toxin into the nerve terminal; or (b) Varespladib may cross the plasma membrane of the nerve terminal, thus being able to bind the toxins inside the motor neuron, blocking their intracellular degenerative action. Antivenom antibodies are not able to reach the toxins once they are internalized in the nerve terminal, hence providing an explanation for the previously described difference in the ability to reverse toxicity between Varespladib and antivenom when treatment is delayed [18]. 

In conclusion, LY315920 and LY333013 are effective at delaying or abrogating lethality in a mouse model of envenoming by *C. d. terrificus*, *B. multicinctus,* and *O. scutellatus*. Moreover, LY315920 is able to reverse paralytic manifestations in severely envenomed mice. These results provide further support to the concept that Varespladib could be a valuable novel therapy in envenomings by neurotoxic snake venoms whose predominant mechanism of action is based on the presynaptic action of neurotoxic PLA_2_s of varying quaternary structure. This proof of concept study did not attempt to optimize dose, dosage form or schedule of administration of these drugs. These and other important issues deserve further investigation. 

## 4. Materials and Methods

### 4.1. Venoms

The venom of *Oxyuranus scutellatus* was obtained from adult specimens collected in Papua New Guinea and supplied by the University of Melbourne. The venom of *Crotalus durissus terrificus* from Brazil was provided by Instituto Butantan. The venom of *Notechis scutatus* was purchased from Venom Supplies Pty Ltd. (Tanunda, Australia) and the venom of *Bungarus multicinctus* was purchased from Latoxan (Portes-lès-Valence, France). These freeze-dried samples were kept at −20 °C until use. For the experiments, venoms were dissolved in 0.12 M NaCl, 0.04 M phosphate, pH 7.2 (PBS) and fresh solutions were prepared for each experiment.

### 4.2. Drugs

LY315920 and LY333013 were provided by Ophirex, Inc., Corte Madera, CA, USA. LY333013 was converted to LY315920 by ChemieTek (Indianapolis, IN, USA). LY315920 was dissolved in PBS for i.v. administration, and LY333013 was dissolved in 8% (*w/v*) gum arabic (Sigma-Aldrich, St. Louis, MO, USA), and was administered orally, as previously described [18]. For both forms of Varespladib, the dose used was 10 mg/kg. This dose was selected on the basis of previous preclinical and clinical data [11,18]. 

### 4.3. Rescue Experiments and Observations on Lethality

#### 4.3.1. Estimation of Subcutaneous Median Lethal Dose (LD_50_)

For all the in vivo experiments done in this study, CD-1 mice weighing 18–20 g were used. For each venom, solutions containing various doses of venom were prepared, using PBS as solvent. Groups of four mice were injected subcutaneously (s.c.) in the thigh, as to simulate a typical snake bite, with 100 µL containing several doses of venoms. The number of dead mice was recorded at 24 h, and LD_50_ was estimated by probit analysis [39]. In the case of *O. scutellatus* venom, the s.c. LD_50_ had been previously determined [23], and the same venom batch of a previous study [18] was used in this work. The protocols of experiments involving the use of mice were approved by the Institutional Committee for the Use and Care of Animals (CICUA) of the University of Costa Rica (CICUA 27-14; 15 July 2014) and met the International Guiding Principles for Biomedical Research involving Animals (CIOMS).

#### 4.3.2. Rescue Experiments

Groups of four mice received a s.c. injection of a dose of the venoms corresponding to three LD_50_s. In the case of venom of *O. scutellatus*, a challenge dose of 12 LD_50_s was also used in order to follow the protocol of a previous study with this venom [18]. Immediately after venom injection, or at various time intervals after envenoming (see details in tables), a dose of 10 mg/kg of either LY315920 (i.v. in the caudal vein in a volume of 200 µL PBS) or LY333013 (orally in a volume of 200 µL gum arabic) was administered. Control mice were injected with venom and with either PBS i.v. or received gum arabic by the oral route instead of the inhibitors. Deaths were recorded at 3 h, 6 h, and 24 h after envenoming.

In the case of the venom of *O. scutellatus*, for which the inhibitors provided full protection during 24 h (see Results), the median inhibitory dose (ID_50_) was estimated. For this, groups of mice received a subcutaneous injection of a dose of venom corresponding to 12 LD_50_s and then, immediately after envenoming, the following doses of LY315920 were administered i.v. in the caudal vein: 10 mg/kg, 5 mg/kg, 2.5 mg/kg, and 1.25 mg/kg, in a volume of 200 µL PBS. Control mice received venom and 200 µL PBS instead of the inhibitor. Deaths were recorded at 24 h and ID_50_ was estimated by probit analysis [39]. In the case of *N. scutatus*, owing to the lack of effect of the inhibitors when administered after venom injection (see Results), experiments in which LY315920 was incubated with venom prior to injection were carried out. A solution of venom (135 µg/mL) and inhibitor (400 µM) was prepared in a volume of 1.0 mL, and incubated at 37 °C for 30 min. Then, aliquots of 100 µL the mixture, containing three LD_50_s of venom, were injected s.c. into a group of four mice. Controls included animals receiving three LD_50_ of venom alone. Lethality was recorded at various time intervals.

#### 4.3.3. Reversal of Paralytic Effects

In order to assess whether the inhibitors are able to reverse the neurotoxic (paralytic) manifestations of envenoming once they have been established, a semi-quantitative toxicity scale for assessing the extent of paralysis was used. In this scale, 0 corresponds to a complete absence of paralysis; 1 corresponds to either paralysis in the hind limbs or respiratory difficulty (forceful respiration); 2 corresponds to both paralysis in the hind limbs and respiratory difficulty; and 3 corresponds to complete paralysis of both hind limbs and fore limbs and respiratory difficulty. When mice were in a degree of envenoming corresponding to grades 2 or 3, a single bolus dose of 10 mg/kg of the inhibitor LY315920, dissolved in 200 µL PBS, was administered i.v. Mice were observed every hour thereafter, for a period of 8 h, and the severity of paralysis was assessed following the described scale.

### 4.4. Inhibitory Effect of Neostigmine

In the case of the venom of *N. scutatus*, in order to assess the possible role of post-synaptically acting α-neurotoxins in lethality, the inhibitory effect of neostigmine was assessed alone or in combination with Varespladib. This drug inhibits acetylcholinesterase, hence increasing the concentration of acetylcholine at the neuromuscular junction. Atropine was administered in conjunction of neostigmine in order to avoid the muscarinic effects of neostigmine. Various groups of four mice received the following treatments: (a) s.c. injection of neostigmine (50 mg/kg) and atropine (50 mg/kg) (Sigma-Aldrich, St. Louis, MO, USA) 5 min before the s.c. injection of three LD_50_s of *N. scutatus* venom; (b) s.c. injection of neostigmine and atropine 5 min before envenoming, and i.v. administration of LY315920 immediately after the s.c. injection of venom; (c) i.v. administration of LY315920 immediately after the s.c. injection of venom; (d) i.v. administration of PBS immediately after s.c. venom injection. Animals were then observed for lethality. 

## Figures and Tables

**Table 1:** Mortality at 3 h of mice injected with *Notechis scutatus* venom and treated with Varespladib (LY315920 or LY333013) (VAR) at various time intervals ^a^.

Treatment	Deaths/Total at 3 h
Venom + PBS	4/4
Venom + VAR (0 min)	4/4
Venom + VAR (60 min)	4/4
Venom + VAR (0 min and 60 min)	4/4

^a^ Groups of four mice received three LD_50_s of venom by the subcutaneous route and then, at the time intervals indicated, they were injected with either PBS i.v. (controls), LY315920 (10 mg/kg) by the i.v. route, or received LY333013 (10 mg/kg) by the oral route. Deaths were recorded at 3 h. Identical results were obtained for treatments with both forms of Varespladib.

**Table 2:** Mortality at 3 h, 6 h, and 24 h of mice injected with *Crotalus d. terrificus* venom and treated with Varespladib (LY315920 or LY333013) (VAR) at various time intervals ^a^.

Treatment	Deaths/Total at 3 h	Deaths/Total at 6 h	Deaths/Total at 24 h
Venom + PBS	4/4		
Venom + VAR (0 min)	0/4	0/4	4/4
Venom + VAR (60 min)	0/4	0/4	4/4
Venom + VAR (0 min and 240 min)	0/4	0/4	4/4
Venom + VAR (60 min and 240 min)	0/4	0/4	4/4

^a^ Groups of four mice received three LD_50_s of venom by the subcutaneous route and then, at the time intervals indicated, they were injected with either PBS i.v. (controls), LY315920 (10 mg/kg) by the i.v. route, or received LY333013 (10 mg/kg) by the oral route. Deaths were recorded at 3 h, 6 h, and 24 h. Identical results were obtained at the various times of observation for treatments with both forms of Varespladib.

**Table 3:** Mortality at 3 h, 6 h, and 24 h of mice injected with *Bungarus multicinctus* venom and treated with Varespladib (LY315920) (VAR) i.v. at various time intervals ^a^.

Treatment	Deaths/Total at 3 h	Deaths/Total at 6 h	Deaths/Total at 24 h
Venom + PBS	4/4		
Venom + VAR (0 min)	0/4	¾	4/4
Venom + VAR (60 min)	0/4	0/4	4/4
Venom + VAR (0 min and 240 min)	0/4	¾	4/4
Venom + VAR (60 min and 240 min)	0/4	0/4	4/4

^a^ Groups of four mice received three LD_50_s of venom by the subcutaneous route and then, at the time intervals indicated, they were injected with either PBS i.v. (controls) or LY315920 (10 mg/kg) by the i.v. route. Deaths were recorded at 3 h, 6 h, and 24 h.

**Table 4:** Mortality at 3 h, 6 h, and 24 h of mice injected with *Bungarus multicinctus* venom and treated with Varespladib (LY333013) (VAR) orally at various time intervals ^a^.

Treatment	Deaths/Total at 3 h	Deaths/Total at 6 h	Deaths/Total at 24 h
Venom + PBS	4/4		
Venom + VAR (0 min)	0/4	0/4	4/4
Venom + VAR (60 min)	0/4	0/4	4/4
Venom + VAR (0 min and 240 min)	0/4	0/4	4/4
Venom + VAR (60 min and 240 min)	0/4	0/4	4/4

^a^ Groups of four mice received three LD_50_s of venom by the subcutaneous route and then, at the time intervals indicated, they were injected with either PBS i.v. (controls), or received LY333013 (10 mg/kg) by the oral route. Deaths were recorded at 3 h, 6 h, and 24 h.

**Table 5:** Mortality at 3 h, 6 h, and 24 h of mice injected with *Oxyuranus scutellatus* venom and treated with Varespladib (LY315920 or LY333013) (VAR) at various time intervals ^a^.

Treatment	Deaths/Total at 3 h	Deaths/Total at 6 h	Deaths/Total at 24 h
Venom + PBS	4/4		
Venom + VAR (0 min)	0/4	0/4	0/4
Venom + VAR (30 min)	0/4	0/4	0/4
Venom + VAR (60 min)	0/4	0/4	1/4 (0/4) ^b^
Venom + VAR (90 min)	0/4	0/4	4/4
Venom + VAR (0 min and 240 min)	0/4	0/4	0/4

^a^ Groups of four mice received 12 LD_50_s of venom by the subcutaneous route and then, at the time intervals indicated, they were injected with either PBS i.v. (controls), LY315920 (10 mg/kg) by the i.v. route, or received LY333013 (10 mg/kg) by the oral route. Deaths were recorded at 3 h, 6 h, and 24 h. When only one deaths/total mice ratio is presented, it means that identical results were obtained when the inhibitor was administered i.v. (LY315920) or orally (LY333013). ^b^ At 24 h, one out of four mice died in the group treated with LY315920 i.v., whereas no mice died in the group treated with LY333013 orally.
